# Comparative efficacy of face-to-face and internet-based cognitive behavior therapy for generalized anxiety disorder: A meta-analysis of randomized controlled trial

**DOI:** 10.3389/fpsyt.2022.832167

**Published:** 2022-07-28

**Authors:** Wenle Zhang, Yun Du, Xiangyun Yang, Encong Wang, Jiexin Fang, Ziqi Liu, Shanqian Wu, Qinqin Liu, Yongdong Hu

**Affiliations:** ^1^Department of Psychological Medicine, Beijing Chao-Yang Hospital, Capital Medical University, Beijing, China; ^2^Department of Clinical Psychology, Capital Medical University, Beijing, China; ^3^The National Clinical Research Center for Mental Disorders and Beijing Key Laboratory of Mental Disorders, Beijing Anding Hospital, Capital Medical University, Beijing, China; ^4^Advanced Innovation Center for Human Brain Protection, Capital Medical University, Beijing, China; ^5^School of Foreign Languages, Shanghai University, Shanghai, China; ^6^Beijing Anding Hospital, Capital Medical University, Beijing, China; ^7^Department of Psychiatry, Capital Medical University, Beijing, China

**Keywords:** cognitive behavior therapy, internet-based cognitive behavior therapy, generalized anxiety disorder, randomized controlled trials, meta-analysis

## Abstract

**Objective:**

The study aimed to ascertain the comparative efficacy of these two forms on reducing anxiety scores of scales in patients with a generalized anxiety disorder (GAD) by examining the available evidence for face-to-face cognitive behavior therapy (CBT) and internet-based cognitive behavior therapy (ICBT). Moreover, this study attempted to determine whether ICBT can obtain similar benefits as CBT for GAD patients during coronavirus disease 2019 (COVID-19) due to the quarantine policy and the requirement of social distance.

**Methods:**

This meta-analysis was registered with the International Prospective Register of Systematic Reviews (PROSPERO) according to the guidelines in the Preferred Reporting Items for Systematic Reviews and Meta-analyses statement (registration number CRD42021241938). Therefore, a meta-analysis of randomized controlled trials (RCTs) examining CBT or ICBT was conducted in this study to treat GAD patients diagnosed with DMS-IV. The researchers searched PubMed, MEDLINE, Embase, PsycINFO, and the Cochrane Database of Systematic Reviews for relevant studies published from 2000 to July 5, 2022. Evidence from RCTs was synthesized by Review Manager 5.4 as mean difference (MD) for change in scores of scales through a random-effects meta-analysis.

**Results:**

A total of 26 trials representing 1,687 participants were pooled. The results demonstrated that ICBT and CBT were very close in the effect size of treating GAD (MD = −2.35 vs. MD = −2.79). Moreover, they still exhibited a similar response (MD = −3.45 vs. MD = −2.91) after studies with active control were removed.

**Conclusion:**

Regarding the treatment of GAD, ICBT can achieve a similar therapeutic effect as CBT and could be CBT's candidate substitute, especially in the COVID-19 pandemic era, since the internet plays a crucial role in handling social space constraints.

**Systematic Review Registration:**

https://www.crd.york.ac.uk/PROSPERO/display_record.php?RecordID=241938, identifier CRD42021241938.

## Introduction

Generalized anxiety disorder (GAD), as a common and disabling illness, is frequently underdiagnosed and undertreated (1). Typical symptoms include excessive anxiety and worry, which occur every day for at least 6 months and are challenging to control for sufferers (2). GAD is characterized by chronic, pervasive anxiety, and worries, accompanied by nonspecific physical symptoms. Additionally, patients with GAD usually experience psychological symptoms such as restlessness, fatigue, difficulty concentrating, irritability, muscle tension, or sleep disturbances (1).

GAD is a chronic, enduring condition. Retrospective accounts suggest that most patients with GAD experience their first episode by 31, among which a quarter experiences it by age 20, with early onset in childhood or adolescence (3). According to representative epidemiologic surveys, the estimated prevalence of GAD in the general population of the United States is 3.1% in the previous year and 5.7% over a patient's lifetime (4). GAD is twice as common in women as in men (5). Carter et al. revealed that GAD is associated with comorbid depression in 70% of cases, any anxiety disorder in over 55%, and somatoform disorders in 48% of cases (6). Consequently, patients with GAD suffer significant mental and physical pain, and are eager to find a way to get rid of these symptoms.

Cognitive behavior therapy (CBT), as a psychotherapeutic treatment, is considered the gold standard for treating GAD (7). CBT better demonstrates how the human mind functions because it is based on an experimental and scientific psychology (8). Hence, CBT for GAD involves cognitive therapy to address worry and cognitive biases and relaxation to handle tension and imaginal exposure to catastrophic images and stressful situations (9). In recent years, the effectiveness of CBT for GAD has been explored by meta-analysis, confirming that CBT is an effective treatment for GAD (10–13). It typically leads to reductions in worry. Such therapy is equal to pharmaceutical treatment and more effective 6 months after study completion (8).

Moreover, CBT may be more effective than some other psychological treatment methods in the longer term, while those were equally effective in the short term (12). Although these results are based on a limited number of studies and should be confirmed in future research, CBT may be preferable over others as the first-line treatment of GAD. Moreover, CBT would have longer-lasting effects compared to usual care.

With the continuous development of network communication, people started to find psychological treatment, removing space and time barriers. Consequently, internet-based cognitive behavior therapy (ICBT) emerged. ICBT is a psychotherapy based on CBT principles and is delivered through the Internet by an individual or program remote from the client (14). ICBT can overcome existing treatment barriers, such as a shortage of trained therapists and ethnic inequalities. The only difference is the format for the delivery of care (15). The main advantages of ICBT are that it is highly accessible, and the required therapist times can be reduced to a fraction of what is necessary for face-to-face CBT (16). ICBT is an effective treatment for GAD across adults and is delivered in routine clinical care. The existing workforce's capacity to manage those seeking help can be improved by continuing to integrate ICBT into existing services, particularly as the population ages (17). ICBT is an effective way to relieve symptoms, improve prognosis, and better GAD patients' life treatment (18–21).

Since November 2019, the outbreak of the novel coronavirus SARS-CoV-2 (coronavirus disease 2019; previously 2019-nCoV) has been spreading worldwide and influencing most people on Earth (22). Measures such as social isolation and home isolation are taken to reduce the virus's spread as much as possible, putting people into a hopeless, nervous, and isolated circumstance. Some studies (23–25) have revealed that the COVID-19 pandemic is inducing additional health problems such as stress, anxiety, depressive symptoms, insomnia, denial, anger, and fear globally, resulting in increased anxiety disorders. This is universally recognized. Therefore, ICBT could be an effective technique to alleviate people's and GAD patients' anxiety symptoms through no face-to-face interaction with each other.

To this end, it is vital to explore whether ICBT has the same effect as CBT from the perspective of evidence-based medicine. There is no related research. In this meta-analysis, the available evidence for face-to-face CBT and web-based CBT (therapist-directed and self-help individual therapy) was examined to determine the effectiveness of both forms in treating GAD and explore whether ICBT could, to some extent, replace CBT as a safer psychotherapy option during COVID-19.

## Methods

### Protocol and registration

This study was registered in Prospero International Prospective Register of Systematic Reviews (PROSPERO) with the registration number CRD42021241938 (https://www.crd.york.ac.uk/PROSPERO/display_record.php?RecordID=241938). It followed the Preferred Reporting Items for Systematic Reviews and Meta-analyses (PRISMA) (26).

### Selection of studies

To identify eligible studies, the researchers searched PubMed, MEDLINE, Embase, PsycINFO, and the Cochrane Database of Systematic Reviews for relevant studies published from 2000 to July 5, 2022. The search terms were ((randomized controlled trial[Filter]) AND ((((((((CBT[Title/Abstract]) OR (cognitive behavior therapy[Title/Abstract])) OR (ICBT[Title/Abstract])) OR (internet-based cognitive behavior therapy[Title/Abstract])) OR (applied relaxation[Title/Abstract])) OR (meta-cognitive therapy[Title/Abstract])) OR (worry exposure[Title/Abstract]) AND (randomized controlled trial[Filter])))) AND (generalized anxiety disorder[Title/Abstract]) OR (GAD[Title/Abstract]). Furthermore, other meta-analysis studies in this field were reviewed, and some original studies that we did not find before were noted.

Both authors selected the studies independently. If there is a discrepancy between the two, they will discuss whether to keep the study or not.

Studies were included in this meta-analysis if they meet the following criteria. (1) Patients were aged 18–65 and met DSM-IV diagnostic criteria for generalized anxiety disorders. (2) Patients were randomly assigned to either CBT/ICBT or control (positive or negative). Specifically, a positive placebo was defined as pills, psychological treatments, and other treatments to improve patients' symptoms; a negative placebo was defined as a waiting list and others that do not take any treatment for the patients. (3) The clinical severity of GAD was assessed through psychometrically sound measures. (4) Studies provided sufficient data of anxiety scores to calculate effect sizes. Studies were excluded if (1) not RCTs; (2) the patients presented other mental disorders; (3) the treatment was combined with other psychotherapy in the CBT/ICBT arm.

### Data collection process and data items

The data was extracted in Microsoft Excel 2019 by two of the authors (Z and D) using a pre-piloted and standardized extraction tool. Details of the region, design, population, diagnosis, sample size, percentage of females, mean age, method, dose, instruments, and comparator were extracted. Moreover, we contacted the authors for additional information when missing data were encountered. Review Manager (RevMan), version 5.4, was employed to generate the risk of bias plots.

### Risk of bias in individual studies

The Cochrane risk of bias tool for randomized controlled trials was adopted to assess the risk of bias within individual trials. Particularly, indicators of selection bias, performance bias, detection bias, attrition bias, and reporting bias were evaluated with the tool (27). Those assessments were completed independently by two reviewers (Z or D). Discussions were held to resolve disagreements between reviewers.

### Meta-analysis

Meta-analyses were conducted on anxiety using RevMan5.4 analysis software. Besides, between-study heterogeneity was assessed using the chi-squared test and I2 statistic. According to the Cochrane guidelines, 0–39%, 40–74%, and 75–100% values were regarded as low, moderate, and high, respectively. Regardless of the heterogeneity test, a random-effects model was employed owing to the inconsistency within the patients, measurement tools, and the characteristics of included studies. Moreover, all the outcomes pooled were continuous in those studies, so as mean differences (MDs) with random-effects meta-analysis. When more than one measurement tool was used in an individual study, all the questionnaires related to generalized anxiety were pooled. The effect sizes were averaged across all outcome measures by $SD=\frac{\sqrt{\left(N_{1}-1\right)SD_{1}^{2\;}+\left(N_{2}-1\right)SD_{2}^{2}+\frac{N_{1\;}N_{2}}{N_{1}+N_{2}}\left(M_{1}^{2}+M_{2}^{2}-2M_{1}M_{2}\right)}}{N_{1}+N_{2}-1}.$ Pre- and post-treatment means and standard deviations (SDs) or the mean and SD of pre- and post-treatment change scores were utilized to calculate the effect sizes.

## Results

### Study selection

**Figure 1:**
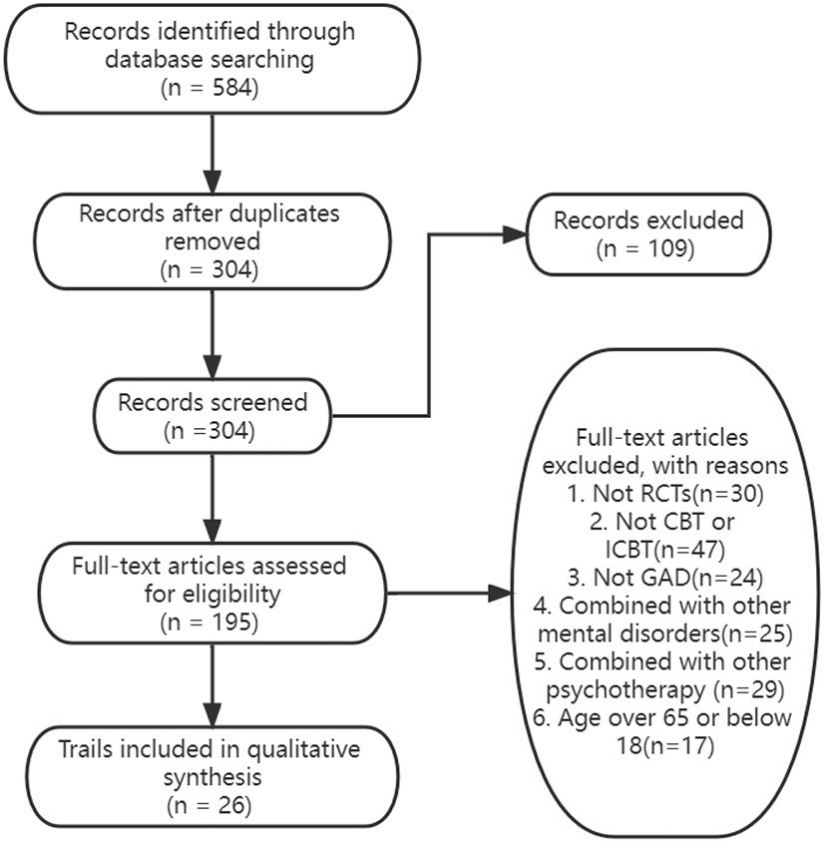
Flowchart of the inclusion of studies.

A total of 584 records were identified using the search strategy (Figure 1). After duplicates were removed, a total of 304 records were screened by title and abstract for potential relevance in this meta-analysis. After title and abstract screening, 109 irrelevant records were excluded, leaving 195 documents for full-text review. After a full-text review, 26 randomized controlled trials for CBT and ICBT (Table 1) satisfied the systematic review and meta-analysis.

**Table 1:** Characteristics of included studies.

**Author/Year**	**Region**	**Diagnose**	**Sample size (experimental/control)**	**Female (%)**	**Mean age**	**Method**	**Dose**	**Instruments**	**Comparator**
Dugas et al. (28)	Canada	GAD/ DSM-IV	65 (33/32)	66%	38.5	CBT	12 sessions, 1 h	CSRADIS	Applied relaxation
Aviram and Westra (29)	Canada	GAD/DSM-IV	35 (17/18)	80%	40.7	CBT	6 sessions, 2 h	PSWQ	MI pre-treatment
Leichsenring et al. (30)	Germany	GAD/ DSM-IV	57 (29/28)	80%	42.5	CBT	30 sessions, 50 min	HARS PSWQ BAI	Short-term psychodynamic
Linden et al. (31)	Germany	GAD/ DSM-IV	72 (36/36)	67%	43.3	CBT	25 sessions, 50 min	HARS STAI-S	Contact control
Hoyer et al. (32)	Germany	GAD/ DSM-IV	49 (18/31)	71%	45.5	Applied relaxation	15 sessions	HAMA PSWQ	Waiting list
Hoyer et al. (32)	Germany	GAD/ DSM-IV	55 (24/31)	77%	45,8	Worry exposure	15 sessions	HAMA PSWQ	Waiting list
Constantino et al. (33)	Canada	GAD/ DSM-IV	85 (42/43)	88%	33.3	CBT	15 sessions, 50 min	PSWQ	MI CBT
Coyne et al. (34)	Canada	GAD/ DSM-IV	85 (42/43)	88%	33.3	CBT	15 sessions, 50 min	PSWQ DASS	MI CBT
Gosselin et al. (35)	Canada	GAD/ DSM-IV	61 (30/31)	59%	50.3	CBT	12 sessions, 90 min	PSWQ	Nonspecific psychological treatment
Newman et al. (36)	United state	GAD/ DSM-IV	83 (40/43)	76%	37.2	CBT+IEP	14 sessions, 50 min	PSWQ CSR HARS STAI-S	I/EP segment
Wells et al. (37)	UK	GAD/ DSM-IV	20 (10/10)	60%	49.05	Metacognitive therapy	8–12 Sessions 50 min	PSWQ BAI	Applied relaxation
Wells et al. (37)	UK	GAD/ DSM-IV	20 (10/10)	60%	49.05	Applied relaxation	8–12 Sessions 50 min	PSWQ BAI	Metacognitive therapy
Heiden et al. (38)	Netherlands	GAD/ DSM-IV	74 (54/20)	73%	35	Metacognitive therapy	14 sessions 45 min	PSWQ	Intolerance-of-uncertainty therapy
Bakhshani et al. (39)	Iran	GAD/ DSM-IV	13 (7/6)	38%	26.5	CBT	8 sessions	BAI HARS DAS	Placebo
Salzer et al. (40)	Canada	GAD/ DSM-IV	57 (29/28)	no details	no details	CBT	mean=28.81(3. 44), 50 min	BAI HADS HARS PSWQ STAI-S	Short-term psychodynamic
Stefan et al. (41)	Romania	GAD/ DSM-IV	71 (23/48)	85%	26.6	CT/BTP	20 sessions, 50 min	GAD-Q-IV PSWQ	ACT
Westra et al. (42)	United state	GAD/ DSM-IV	76 (38/38)	67%	41.9	CBT	14 sessions, 50 min	PSWQ DASS-A	MI pre-treatment
Ladouceur et al. (43)	Canada	GAD/DSM-IV	26 (12/14)	77%	39.7	CBT	16 sessions, 1 h	ADIS-IV PSWQ BAI	Waiting list
Titov et al. (44)	Australia	GAD/DSM-IV	45 (21/24)	no details	no details	ICBT	6 sessions	GAD-7 PSWQ	Waiting list
Andersson et al. (45)	Netherlands	GAD/DSM-IV	54 (27/27)	76%	42.02	ICBT	8 sessions	PSWQ GAD-Q-IV STAI-state STAI-trait BAI	Waiting list
Paxling et al. (46)	Sweden	GAD/DSM-IV	89 (44/45)	79.80%	39.3	ICBT	8 sessions	PSWQ GAD-Q-IV STAI-S STAI-T BAI	Waiting list
Hadjistavroppulos et al. (47)	Canada	GAD/DSM-IV	174 (91/83)	78.70%	38.3	ICBT	5 sessions	GAD-7	Optional weekly therapist support
Robinson et al. (48)	Australia	GAD/DSM-IV	98 (50/48)	67%	44.84	TA-ICBT	6 sessions	PSWQ GAD-7	Delay treatment
Robinson et al. (48)	Australia	GAD/DSM-IV	95 (47/48)	71.60%	45.52	CA-ICBT	6 sessions	PSWQ GAD-7	Delay treatment
Robichaud et al. (49)	Canada	GAD/DSM-IV	63 (32/31)	87.3%	35.19	ICBT	8 sessions	GAD-7	Waiting list
Christensen et al. (50)	Australia	GAD/DSM-IV	222 (111/111)	no details	25.85	ICBT	10 sessions	GAD-7 PSWQ	Placebo

*GAD: generalized anxiety disorder; CBT: cognitive behavior therapy; ICBT: internet-based cognitive behavior therapy; DSM: the diagnostic and statistical manual of mental disorders; CSRADIS 9- point (0 to 8): Clinician's Severity Rating of the Anxiety Disorders Interview Schedule for DSM-IV; PSWQ: Penn State Worry Questionnaire; HARS: Hamilton Anxiety Rating Scale; BAI: Beck Anxiety Inventory; DASS: Depression Anxiety Stress Scales, 21-item version; CRS Clinician's Severity Rating; GAD-Q-IV: generalized anxiety disorder questionnaire; HADS: Hospital Anxiety and Depression Scale; GAD-7: Generalized Anxiety Disorder 7-Item; MI: motivational interview; ACT: acceptance and commitment therapy; I/EP: segment interpersonal/emotional processing segment*.

### Characteristics of studies

Details of the characteristics of included studies are listed in Table 1. All the studies included were published in English, and most of the researchers are from the Americas, Europe, and Australia. All the included studies were designed for patients with GAD diagnosed by the diagnostic and statistical manual of mental disorders fourth edition (DSM-IV). Anxiety symptoms before and after intervention were measured by some of the scales of anxiety. Specifically, most of the studies used The Penn State Worry Questionnaire (PSWQ). Other scales such as the Generalized Anxiety Disorder 7-Item (GAD-7) Scale and State-trait anxiety inventory (STAI) were also adopted in those studies. More than 10 sessions in the intervention period were provided for 14 out of 26 trials, and at least five sessions were offered for all the studies.

### Overview of results of the pairwise meta-analysis

**Figure 2:**
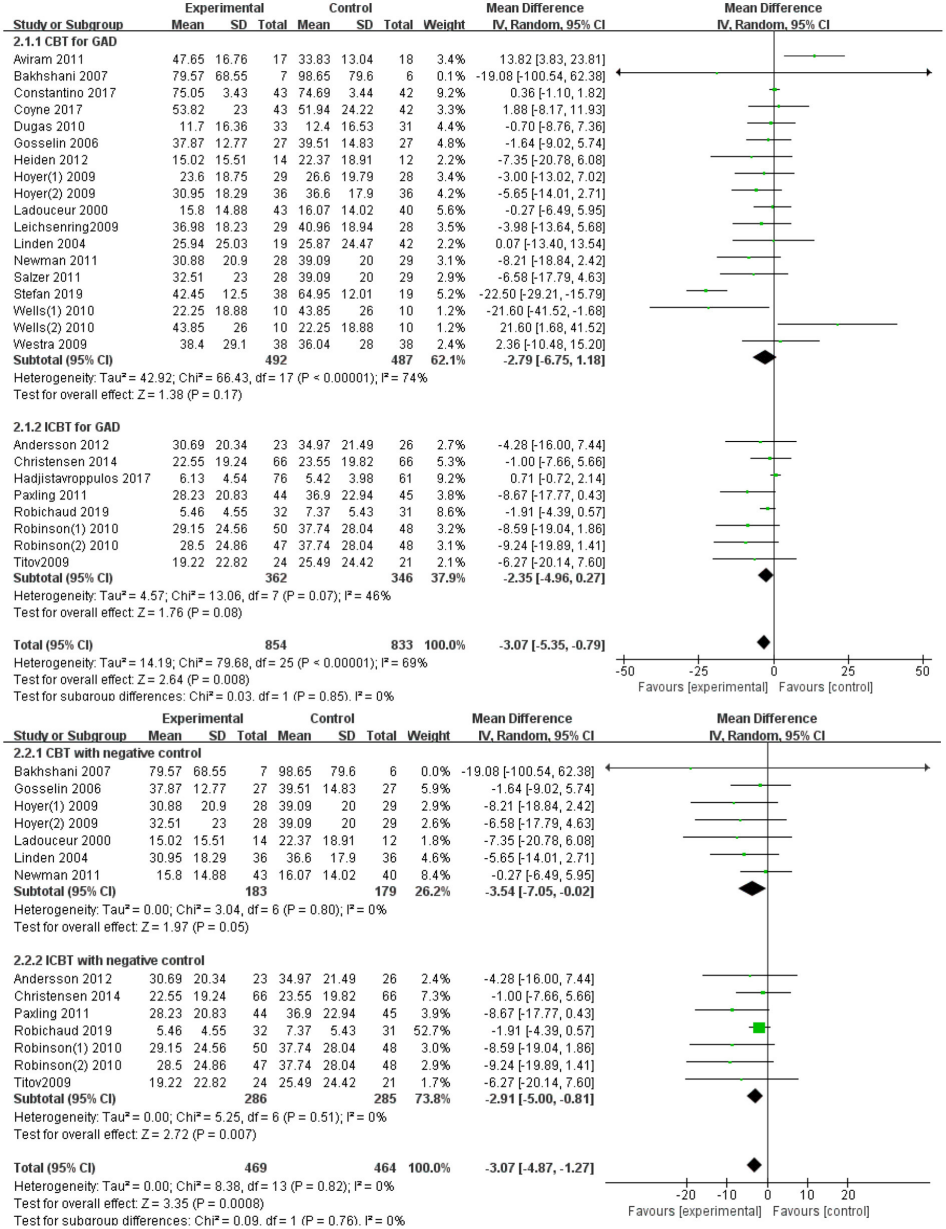
Forest plots.

Effect sizes and 95% CI for anxiety symptoms for different treatments are presented in Figure 2. The forest plot is from top to bottom: (1) subgroup meta-analysis of anxiety scores in the treatment of GAD with CBT and ICBT; (2) subgroup meta-analysis of anxiety scores in the treatment of GAD with CBT and ICBT after removed trails with active comparators.

**Figure 3:**
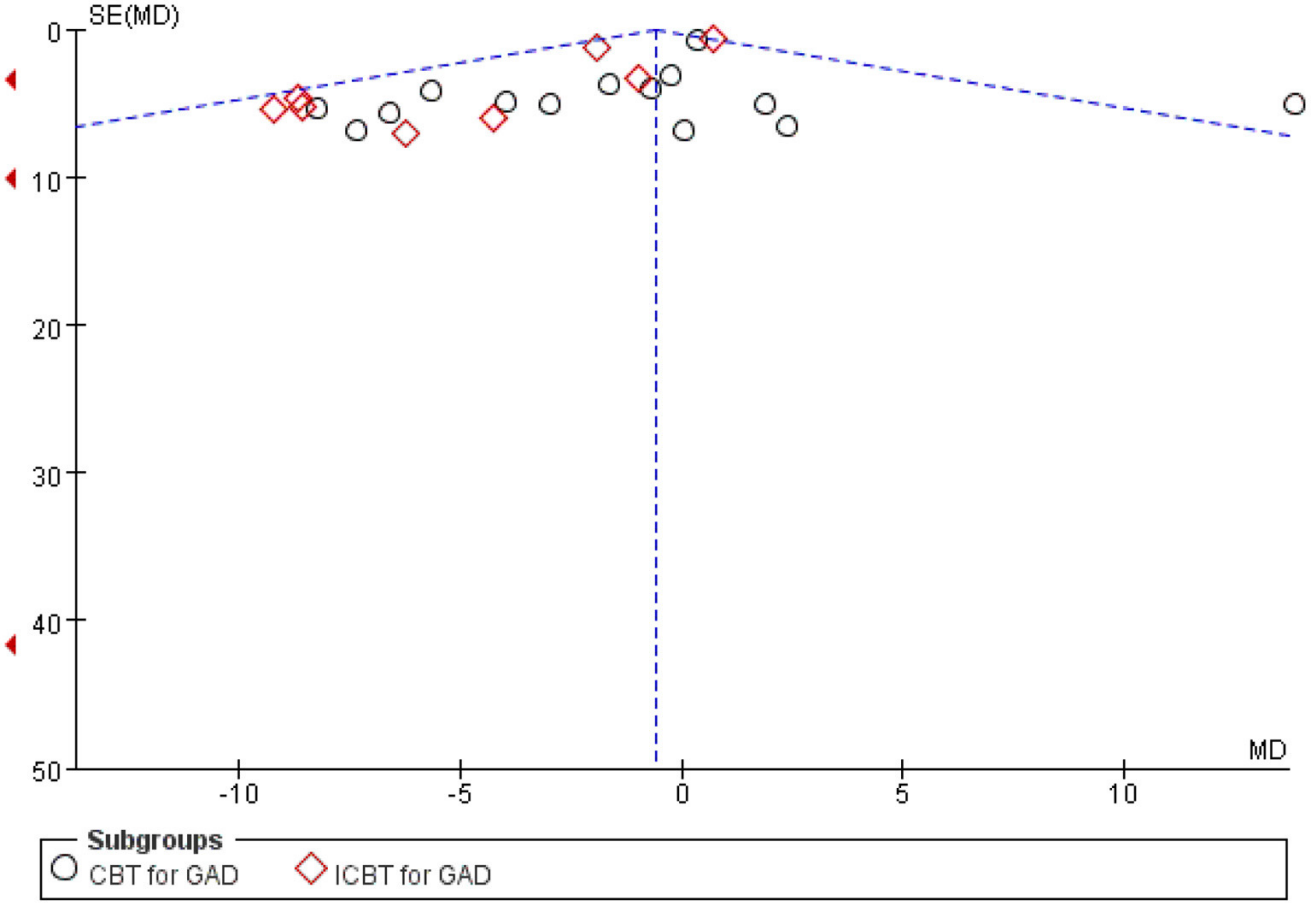
Funnel plot.

**Figure 4:**
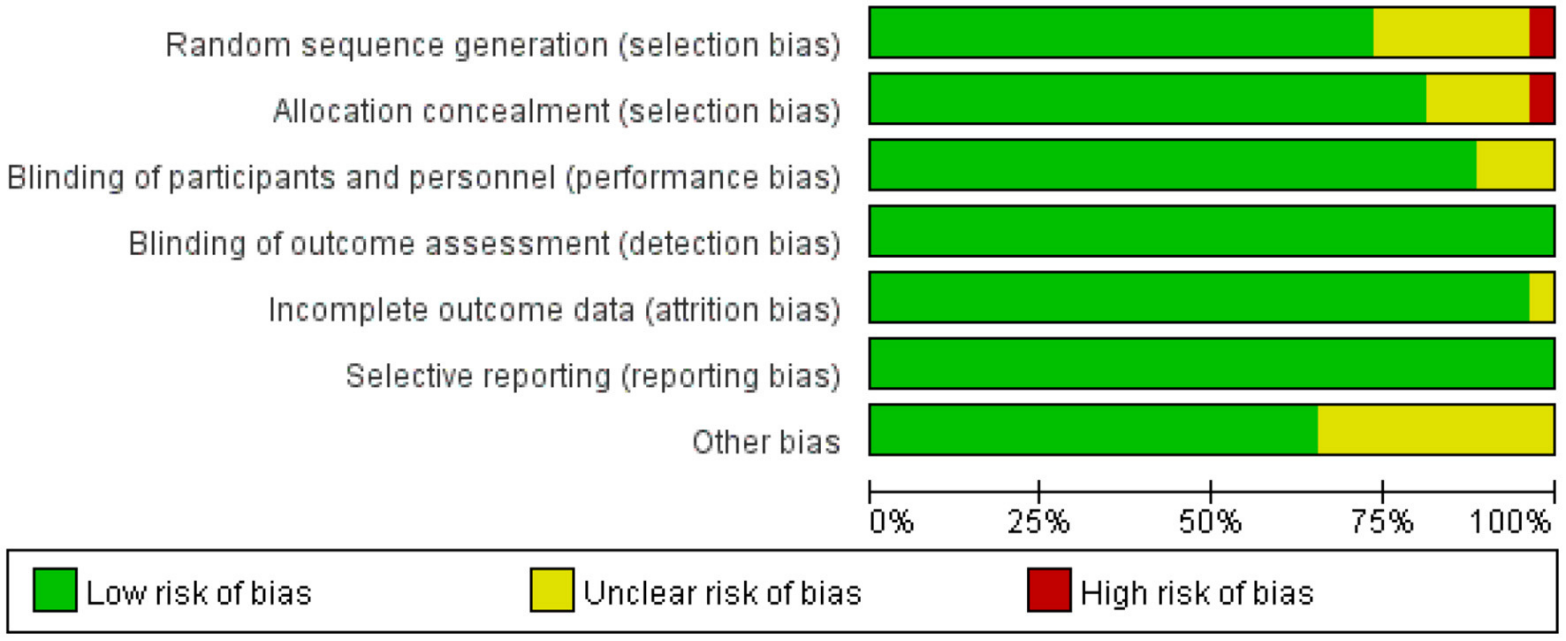
Risk of bias graph.

All trials reported anxiety rating scores, and it was considered the primary outcome. All the results in individual trials were combined since most of the trials pooled were used at least one measure tool. There were 18 trails in the face-to-face CBT group and eight trails in the internet-based CBT group. Figures 3, 4 illustrate a summary of the pooled meta-analysis outcomes. CBT demonstrated a greater improvement compared with ICBT (MD = −2.79, 95%CI: −6.75; 1.18 vs. MD = −2.35, 95%CI: −4.96; 0.27).

In the ICBT Group, seven trials identified the control group on a waiting list, suggesting that eventually all of these patients were treated. Nonetheless, the control group was always negative during the study period. However, part of the CBT study was an active control group; some of the control groups adopted drug therapy, and some used other forms of psychological treatment. This difference would be induced by the following factors. Patients with CBT may be either inpatients or outpatients, and few researchers utilize active controls because of the delay in patient recovery. However, patients on ICBT may be recruited from the community, and the wait-list approach is more appropriate in milder cases. Thus, the trails with active comparators were removed and analyzed again to draw a new forest graph. ICBT still exerted a similar effect compared to CBT for treating GAD (MD = −2.91, 95%CI: −5.00; −0.81 vs. MD = −3.54, 95%CI: −7.05; −0.02).

### Publication bias and risk of bias

**Figure 5:**
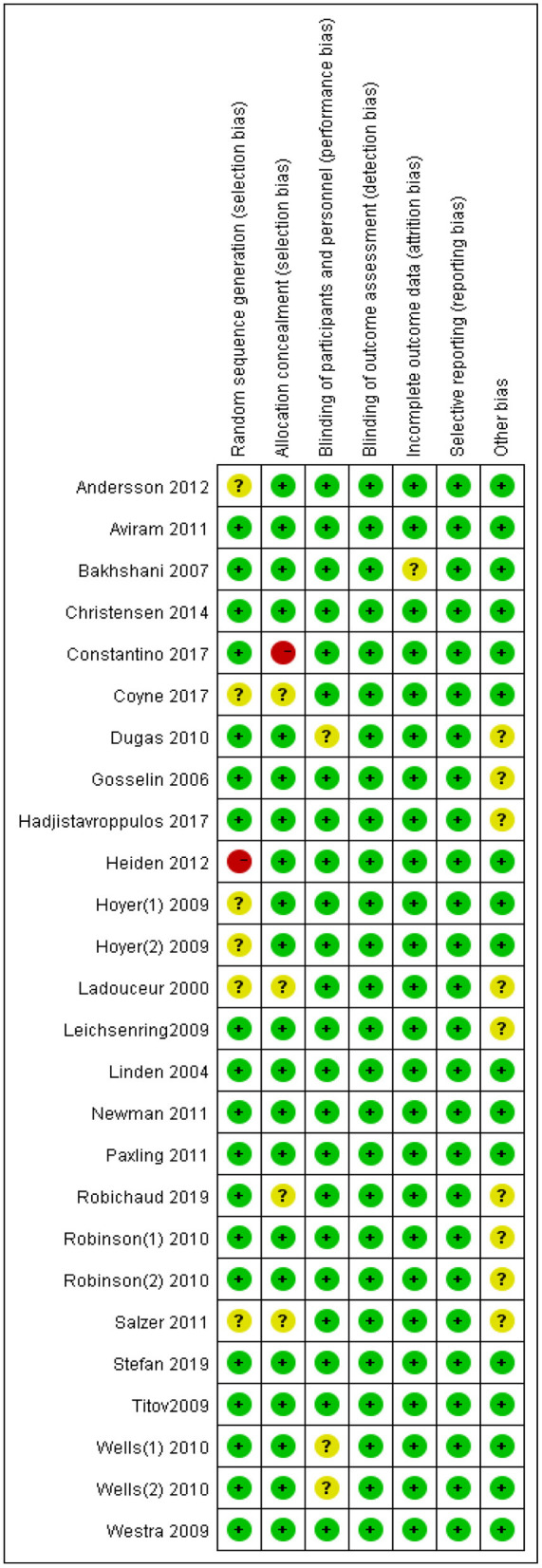
Risk of bias summary.

The results of the publication bias assessments are depicted in Figure 3. No significant publication bias in anxiety rating scores was observed in the funnel plot. The overall quality of the 26 trials included in the meta-analysis was high, and only a handful of studies had any “high risk” domains (Figures 4, 5).

## Discussions

Many factors affect the changes in patients' anxiety symptoms, and randomized controlled trials could be the most effective method to investigate the therapeutic effects of CBT and ICBT for treating GAD. Therefore, the effects of CBT and ICBT in the treatment of GAD were compared in this meta-analysis only based on randomized controlled studies to obtain more accurate and objective conclusions. In this paper, 26 randomized controlled trials were reviewed, including 18 in the CBT group and 8 in the ICBT group. The difference in the number of studies between the two groups may be induced by the following factors. First, ICBT is the type of internet-based therapy emerging in recent years, and most studies may not be conducted in hospitals. As a result, RCTs will be fewer. Second, all studies of patients with GAD were identified using the DSM-IV system, which was a step to minimize bias. Moreover, a literature search suggested that the vast majority of studies used this system. Besides, 1,687 patients were randomly assigned to the CBT, ICBT, and placebo treatment groups to treat GAD. The results revealed that the CBT and ICBT groups were significantly better than the placebo in comparing anxiety levels before and after treatment. Compared with placebo, CBT was a treatment method with more significant benefits for anxiety-related disorders. The unique effect of CBT has gone beyond the scope of disease symptoms while presenting a bearing on the lives of patients as a positive benefit.

As unveiled by reviewing the previous studies in the field of psychiatry (51, 52), clinician-guided ICBT seems to work as effective as face-to-face CBT for some disorders. Moreover, ICBT has been discovered to be effective for somatic conditions, such as tinnitus and irritable bowel syndrome (53). A Cochrane review (54) of ICBT for anxiety disorders suggested that it is effective, with a standardized mean difference against no treatment control of 1.06, which is a large effect. Furthermore, therapist-guided ICBT may be as effective as face-to-face CBT, consistent with the conclusion of our study. Concerning GAD only, positive results of ICBT for GAD have been investigated in a few controlled studies including long-term follow-up after treatment completion. However, there are no comparative trials against face-to-face CBT and no meta-analysis related to GAD treated by ICBT compared with CBT.

Therefore, the comparative effects of CBT and ICBT for GAD were explored in this meta-analysis. Many studies have examined the effect of those two types of psychological treatments for GAD in adults. Generally, CBT and ICBT are more effective than waiting list control groups or even active control groups on worries, anxiety, and depression, regardless of whether effects were measured with which kind of instruments.

Specifically, our first goal of this meta-analysis was to determine whether ICBT can obtain similar benefits to CBT in treating GAD. The comparative effects were tested on other mental disorders, such as psychiatric and somatic disorders (55). In other words, ICBT and face-to-face treatment are similar and produce equivalent overall effects, in line with our results. Moreover, therapist-supported ICBT is more efficacious than a waiting list, attention, information, or online discussion groups only, and there may not be a significant difference in outcome between unguided CBT and therapist-supported ICBT (54). The evidence suggests that therapist-supported ICBT may not be significantly different from face-to-face CBT in reducing anxiety, similar to our conclusion. Some other studies (16, 56, 57) present the treatment format and review the evidence for mood and anxiety disorders, concluding that ICBT is becoming one of the most evidence-based forms of psychological treatment. The previous studies and our study uncover that ICBT can obtain similar benefits like CBT for treating GAD and even other mental disorders.

Nevertheless, patients treated with ICBT may have milder symptoms. Hence, the response to ICBT may be better, and patients who can complete an entire course of ICBT treatment, their desire for, and confidence in healing, were also better. This may explain why ICBT is slightly better than CBT in the overall effect.

This study aimed to reveal whether ICBT can replace CBT during the COVID-19 pandemic period for our second goal. The coronavirus disease 2019 (COVID-19) outbreak, which has caused >46 million confirmed infections and >1.2 million coronavirus-related deaths, is one of the most devastating worldwide crises in recent years (58). Social distancing is the most visible public health response and effective mothed to the COVID-19 pandemic (59–61). Thus, a series of mandatory actions have been taken by the municipal and provincial governments supported by the central government, such as measures to restrict travel across cities, case detection and contact tracing, quarantine, guidance and information to the public, and detection kit development (62). Consequently, face-to-face psychological treatment may increase the risk of infection, and it is imperative to determine whether internet-based can replace face-to-face psychological treatment. According to the meta-analysis results, the overall effect of ICBT is better than CBT. Hence, ICBT can replace CBT during the COVID-19 pandemic and even become the primary psychological method in the future due to its characteristics such as convenience and economics.

Although ICBT has many advantages, it has some limitations. First, patients are required to have a higher level of education for self-service ICBT since the instructions are mainly provided in text form. Second, participants are generally well-educated in many studies. This would be no different from typical psychotherapeutic studies while limiting the possibility of extending the research results to conventional medical settings. Third, few studies adopt reliable attention control conditions, though direct comparison studies with face-to-face CBT exhibit small differences in results, raising questions about the specificity of the results.

Despite these limitations, research and clinical implementation studies are promising and could boost the chances of obtaining evidence-based psychotherapy. Many questions remain to be answered. Nevertheless, clinicians will increasingly combine their routine services with ICBT as a supplement or alternative for certain patients considering that information technology may be continuously developed.

This study also presents several limitations related to the included studies, such as the small number of studies using other than waiting list control groups and the lack of follow-up measurements. Besides, the measurement tools used in those trials are so different that we could not find the same one in 26 trials. As a result, those results had to be combined, and this may influence the final result.

Concurrently, the RCT studies of ICBT treatment for GAD are few and all focus on the years after 2000, while the study of CBT is more numerous, causing bias in the results. To minimize this bias, we referred to the same type of study and limited the time of publication to 2000. ICBT emerged after 2000 and was of concern to researchers, whereas CBT has been widely studied since earlier times. Hence, studies from the same time period were compared to minimize the results bias.

## Conclusion

Despite the limitations of this meta-analysis, this is the first systematic review and meta-analysis for RCTs that have compared the performance of CBT to ICBT for the treatment of GAD. The final MDs of CBT and ICBT are close, suggesting that the effect sizes of ICBT and CBT were similar in anxiety score reduction. This verified the efficacy of the internet-based CBT treatment. Nevertheless, the results demonstrated that ICBT has an equal treatment effect with CBT and can replace CBT during the COVID-19 pandemic as a safer method.

## Data availability statement

The original contributions presented in the study are included in the article/supplementary material, further inquiries can be directed to the corresponding author.

## Author contributions

YH reviewed the manuscript. WZ designed the research, searched the literature, extracted the data, and wrote the manuscript. YD searched the literature and extracted the data. SW and QL checked and analyzed the data. ZL reviewed and checked the language of this manuscript. XY, EW, and JF reviewed the manuscript and made changes suggestions. All authors read and approved the manuscript.

## Conflict of interest

The authors declare that the research was conducted in the absence of any commercial or financial relationships that could be construed as a potential conflict of interest.

## Publisher's note

All claims expressed in this article are solely those of the authors and do not necessarily represent those of their affiliated organizations, or those of the publisher, the editors and the reviewers. Any product that may be evaluated in this article, or claim that may be made by its manufacturer, is not guaranteed or endorsed by the publisher.
